# Research on Classroom Emotion Recognition Algorithm Based on Visual Emotion Classification

**DOI:** 10.1155/2022/6453499

**Published:** 2022-08-08

**Authors:** Qinying Yuan

**Affiliations:** Xi'an Jiaotong University, Shaanxi, Xi'an 710049, China

## Abstract

In this paper, we construct a classroom emotion recognition algorithm by classifying visual emotions for improving the quality of classroom teaching. We assign weights to the training images through an attention mechanism network and then add a designed loss function so that it can focus on the feature parts of face images that are not obscured and can characterize the target emotion, thus improving the accuracy of facial emotion recognition under obscuration. Analyze the salient expression features of classroom students and establish a classification criteria and criteria library. The videos of classroom students' facial expressions are collected, a multi-task convolutional neural network (MTCNN) is used for face detection and image segmentation, and the ones with better feature morphology are selected to build a standard database. A visual motion analysis method with the fusion of overall and local features of the image is proposed. To validate the effectiveness of the designed MTCNN model, two mainstream classification networks, VGG16 and ResNet18, were tested and compared with MTCNN by training on RAF-DB, masked dataset, and the classroom dataset constructed in this paper, and the final accuracy after training was 78.26% and 75.03% for ResNet18 and VGG16, respectively. The results show that the MTCNN proposed in this paper has a better recognition effect. The test results of the loss function also show that it can effectively improve the recognition accuracy, and the MTCNN model has an accuracy of 93.53% for recognizing students' facial emotions. Finally, the dataset is extended with the training method of expression features, and the experimental study shows that the method performs well and can carry out recognition effectively.

## 1. Introduction

Human emotions are closely related to behavior. Emotions originate from objective facts and are the key to determining the quality of human daily life, and they are related to human needs. When human needs are met, humans tend to generate positive emotions and show positive behaviors [1]. The classroom teaching environment is different from the one-on-one online tutoring teaching environment and offline tutoring teaching environment because the number of students is large, and the teacher cannot pay attention to each student's emotional condition and always give feedback while considering the course schedule [2]. The emotional state of students in the learning process may have a positive or negative impact on the overall learning results, which leads to students in a depressed mood because they cannot adjust their state in time to reduce enthusiasm for learning, affecting the efficiency of the entire course, which is undoubtedly half the effort. The pursuit of teaching efficiency is the essential characteristic of teaching, which is also an important goal of the current curriculum reform and an inevitable requirement for the internal development of education [3]. In the process of classroom teaching, teachers hope to understand students' emotional changes and learning status in real time, adjust the course progress in time, and ensure that students can grasp the content taught in a timely and comprehensive manner. According to Mehrabian, 55% of emotional information is expressed through facial expressions [4]. Therefore, facial expression recognition can determine students' emotional state and provide timely feedback to the teacher, so that the teacher can judge the current classroom students′ emotional change in real time, make a timely course adjustment, activate the classroom atmosphere, mobilize students′ learning enthusiasm, and improve the overall classroom learning efficiency.

Sentiment analysis, also called opinion mining, is the process of processing and analyzing user-generated content to study the emotions, opinions, and attitudes expressed by users and to judge or evaluate them. Sentiment, as an important attribute of humans in social behavior, represents an individual's viewpoint and attitude toward a specific goal and has a great impact on all aspects of human life such as cognition, socialization, decision-making, and reasoning [5]. The current work on sentiment analysis in social media is mainly focused on text analysis, and a lot of research results have been achieved, but the research on sentiment analysis based on visual content such as images and videos is still very limited. As the saying goes, “a picture is worth a thousand words,” compared with textual content, images and video content are simpler and more intuitive; especially, with the development of multimedia technology and the popularity of photographic devices, more users tend to use visual content such as images to express their views and emotions, and the amount of visual content in social media is therefore growing. The amount of visual content in social media is growing as a result [3]. The identification and retrieval of these visual contents have been widely used in various fields; however, the emotions and opinions of users conveyed by these visual contents largely reflect their behavioral preferences and needs, so it is also important to deeply explore the attitudes and emotions in them for public opinion monitoring and enterprise decision-making. Although the emotions embodied in visual contents are complex and subjective, there are commonalities in human cognition of emotions, which is the basis of visual sentiment analysis [6]. Traditional research on visual sentiment analysis mainly uses image low-level visual features for sentiment analysis, such as color features, texture features, and contour features. However, these algorithms are not sufficient to bridge the huge semantic gap between low-level visual features and high-level sentiment semantics of images, so the effect of sentiment classification is not satisfactory.

In recent years, artificial intelligence plays an increasingly important role in education, and AI and deep learning-related technologies (such as image recognition, semantic recognition, and speech recognition) are gradually implemented into specific fields, providing technical support for the development of adaptive education. The conference pointed out that one of the applications of AI in education is real-time monitoring of students′ learning expressions, learning postures, and other valuable information [7]. In traditional classrooms, the number of students is large and the teacher needs to control the teaching pace, so it is difficult to pay attention to each student′s emotional state and make corresponding adjustments to feedback during the classroom teaching process, which makes students unable to adjust their state in a time when they are depressed, thus reducing their enthusiasm for learning, and affecting teaching efficiency, which is undoubtedly half the effort [8]. The traditional classroom evaluation methods, such as postclass conversations and questionnaires, are too delayed and cannot help teachers make timely adjustments. Therefore, how to provide teachers with timely and effective feedback on students′ emotional states in class has become an urgent problem in classroom teaching. This paper constructs an algorithmic model for classroom emotion recognition with the help of artificial intelligence, which has positive significance for technology to focus on students′ classroom emotions and improve the quality of classroom teaching.

## 2. Related Works

Traditional visual sentiment analysis methods mainly use knowledge from fields such as visual cognition and psychology to extract underlying visual features such as color, texture, and shape of images and perform sentiment analysis through statistical methods [9]. The University of Chile has developed a texture retrieval system based on soft computing techniques. By studying the human subjective psychological perception of texture, the system obtains qualitative descriptions of texture consisting of 12 adjectives, which can be further used for texture querying, and evaluates 100 textures by selected adjectives to associate texture features with these subjective qualitative descriptions through neural networks. Yin et al. achieved high accuracy by detecting the shape of straight lines in images, further extracting the straight-line orientation histogram features of the images, and fusing other features for sentiment recognition [10]. However, these methods extract features based on simple statistical methods and do not consider the human emotion element. Hossain combines human emotion and art painting theory based on psychological principles, thus achieving emotion category labeling of art images [7]. Since traditional visual sentiment analysis methods are not sufficient to overcome the semantic gap problem, some researchers have started to construct intermediate semantic representations around the semantic content of images to better express the sentiment of images. The most representative of these works is the approach proposed by Kim H H et al. which is by designing an adjective noun pair (ANP) and using the ANPs corresponding to different images as intermediate representations of visual sentiment, e.g., beautiful flower, lovely dog, etc. [11]. However, there are still some problems in the practical application of the visual emotion ontology library VSO. First, the ANPs in VSO can only describe the visual concepts in the images and cannot identify which ANP is highly related to the main emotion in the images; second, the images in social networks have different themes, which can be divided by themes, and a theme usually contains many related images. However, the VSO model cannot fuse the sentiment information of multiple related images under the same topic and thus cannot select more representative ANPs to describe the sentiment of images. To solve the above problems, Li et al. proposed a visual emotion-based topic model, which tries to enhance the understanding of visual semantic objects in images and their emotions through topics in social networks [12].

A good teacher can accurately grasp classroom emotions to improve students′ attention, make them feel happy and satisfied, and eventually make them enjoy their lessons, while a teacher who is not good at grasping classroom emotions may cause the classroom to fall into a boring narrative, students′ attention is not focused, and learning is inefficient. Scholars have done enough research on classroom emotions and have deeply analyzed teachers′ and students′ behaviors and emotional expressions in the classroom and what kind of teaching effect the classroom will have after these expressions. Professor Luo H argues that the affective dimension can be seen as a continuum of hierarchical order and describes emotions not only as simple or complex, figurative, or abstract interpretations but also as characteristics of control from the unconscious to the internal and external, which can be called internalization [13]. Rani et al. proposed a classification theory of educational goals, for which the goal classification system needs to be divided according to the emotions in it, and the dimensions of its division are mainly reflected in five levels, namely, acceptance, reaction, value judgment, organization, and characterization of value and value complexes, where each of the levels has its emotional meaning, and which also has a sublevel corresponding to that level [14]. For emotion recognition in a classroom environment, Zeng et al. obtained facial features through the AAM model combined with constrained local model (CLM) and classified classroom emotion categories into five types: listening, doubt, understanding, resistance, and disdain, and based on Izard′s maximum discriminative facial action coding system, judged by facial offset angle, eye and eyebrow angle, and mouth angle [15]. The five expression states were judged by the changes in the characteristics of the three parts of the face, the angle between the eyes and the eyebrows, and the corner of the mouth.

In classroom teaching, teachers should pay attention to students′ learning emotions and promptly adjust their teaching strategies based on the feedback from expressions to mobilize students' positive learning emotions. Scholars have conducted many studies on classroom teaching and learning, which have provided comprehensive and in-depth analyses of classroom teaching and learning processes from many different perspectives, and have thus contributed to further development of teaching and learning. Since 2000, the integration of computer sentiment analysis and educational teaching has been greatly promoted. Researchers have used video analysis to understand learners′ emotional states, providing teachers with timely feedback so that they can revise course content, adjust course difficulty, select teaching methods, and control the teaching schedule, greatly promoting changes in the teaching model and improving teaching quality [16]. The biggest problem of emotion recognition research in a classroom teaching environment is the need to detect the unique emotion category for the unique environment, there is a serious lack of publicly available emotion datasets for the classroom environment, and due to the large change in students′ posture and orientation in the classroom environment, the existence of such actions as lowering the head and turning sideways will lead to incomplete face pictures and coupled with the influence of background, illumination, and occlusion, the available face images are less. How to recognize the emotion and achieve a high accuracy rate despite the presence of facial occlusion is also a focus of the research.

## 3. Visual Emotion Classification Classroom Emotion Dataset Construction

In classroom teaching, students′ expressions and behaviors can greatly express their mental and emotional states. For courses they are interested in or like, they tend to show happy, focused, and positive emotions, with facial expressions such as smiles and concentration. For courses that they do not like or even dislike, they tend to show negative emotions such as resistance and disdain, and their faces are accompanied by negative expressions such as frowns. These negative emotions are partly caused by the difficulty of the course and the difficulty of adapting to the teacher′s teaching style, while another part is caused by the students′ psychological problems. Therefore, the identification and analysis of students′ emotions in the classroom environment can not only help teachers to make better adjustments to the course teaching but also help to detect whether students have psychological problems in time and provide timely psychological counseling and other treatments for students with psychological problems, to promote the overall development of students′ body and mind. This paper realizes the prediction of dimensional emotional data based on related networks such as recurrent neural network and finally uses the quantitative algorithm of emotional eigenvalues to calculate emotional intensity, which provides a database for the system to perform adaptive adjustment.

Get the students' dynamics in class in real time through the camera, then extract features through image processing, machine learning, deep learning, and other technologies, classify emotions, and feed back the real-time emotional status to the teacher in time. The teacher makes corresponding adjustments according to the students' current emotional status. When students are depressed, adjust the teaching progress, activate the classroom atmosphere, and improve students' learning motivation. When students are generally in a low mood, the teacher will adjust the teaching schedule to improve students′ learning enthusiasm and efficiency; when students are generally in a high mood, the teaching schedule can be accelerated to further improve the teaching efficiency of the course.

The convolutional neural network is a special kind of feed-forward neural network with numerous neurons in its structure, and these neurons come with weights and biases. It uses local connections to avoid the problem of redundant data caused by full connections [17]. In the convolutional neural network, its local connectivity can effectively reduce the number of parameters in the network, which can reduce the dependence of the model on a large amount of training data. In simple terms, for a two-dimensional image data, the color image is input to the neural network by default, and then the size of the color image is set to the standard data format: depths × height × width. Multi-task convolutional neural network (MTCNN) provides a multi-task cascade framework for face detection, which is characterized by two steps of face detection and key point localization being performed simultaneously. When an arbitrary image is given, MTCNN scales it to different scales to form an image pyramid for scale invariance.

(1) $$\begin{array}{c} p\left(v,h|\theta \right)=\ell^{E\left(\right)v,z\left(\theta \right)}, \end{array}$$

*m* is the number of neurons in the hidden layer, *n* is the number of neurons in the visible layer, and *W*_*mn*_ is the weight matrix between them, *v*=[*v*_1_, *v*_2_,…, *v*_*n*_] is the state of the visible layer, and *h*=[*h*_1_, *h*_2_,…, *h*_*m*_] is the state of the hidden layer. For example, if we assume that there are *n* visible units in the visible layer and *m* hidden units in the hidden layer, the energy function between the nodes in the visible layer and the nodes in the hidden layer can be expressed as follows when the values of both visible and hidden units are 0 or 1.

(2) $$\begin{array}{c} E\left(v,h|\theta \right)=\sum_{i=1}a_{i}v_{i}+\sum_{j=1}^{n}v_{i}b_{j}w_{ij}. \end{array}$$

In the formula, *v*_*i*_ represents the state of the ith unit of the visible layer, *h*_*j*_ represents the state of the jth unit of the hidden layer, *w*_*ij*_ represents the connection weight between *v*_*i*_ and *h*_*j*_, and *a*_*i*_ and *b*_*j*_ represent *v*_*i*_ and the bias value of *h*_*j*_.

A deep neural network (DNN) is also a neural network, but it has multiple layers of perceptron and multiple hidden layers and consists of a series of Boltzmann machines (RBMs) stacked and trained layer by layer. The lower layers represent the original information of data features, while the higher layers represent the attribute categories of data, so its learning process proceeds from the lower to the higher layers. In the learning process, the higher layers will continuously acquire deep abstract features, which is the essential feature of deep learning data. According to the above research status, although researchers have proposed many feature extraction methods and recognition classification methods for pictures, there are still some problems to be solved if we want to realize a system of expression recognition with high accuracy and in real time: one is the problem of how to perform multi-person face detection in real time; the other is the problem that some external factors and similarity between faces under natural conditions can affect the accuracy rate.

(3) $$\begin{array}{c} p\left(h_{i}=1|v\right)=\delta \left(\sum_{i=1}a_{i}w_{ij}-b_{j}\right) \end{array}$$

In 1976, Paul Ekman created the facial action coding system (FACS), which divided the face into independent and related AU—action unit—based on the anatomical characteristics of different muscles of the face. The FACS provides a detailed classification and elaboration of motor units and expression composition, which has become the main theoretical basis for later research, and many classical algorithms or expression databases are based on the FACS. The classroom expression status categories should indicate whether the learners are interested in the content, whether they agree with the teacher′s teaching style, and whether they are comfortable with the pace of knowledge taught. Teachers can use this as a basis for adjusting the content and teaching process and providing timely emotional compensation. Teaching observers can use this to evaluate the quality of teaching, reflect on teaching activities, and make changes. Since the focus and application scenarios of classroom expressions are different, they are both the same and different from the common expressions.

**Figure 1:**
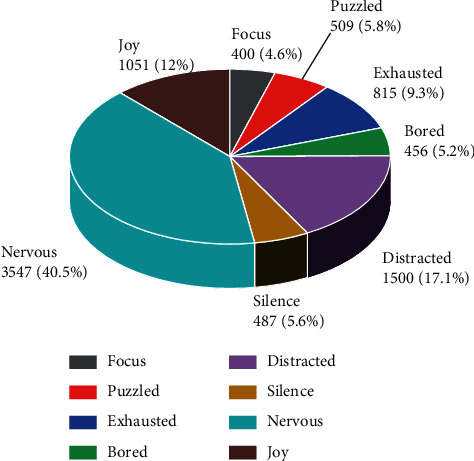
Number of samples collected for each type of emotion.

The discrete emotion description model describes emotions into discrete categories, such as happy, angry, and sad. Ekman, an American psychologist, proposed 6 basic human emotions: happy, angry, surprised, sad, disgusted, and afraid. The Chinese emotion corpus of the Chinese Academy of Sciences Institute of Automation (CASIA) also basically follows these six basic emotion categories, except that disgust is replaced with neutrality according to the corpus data [18]. The present dataset refers to a wide range of human emotions as well as the actual emotional performance in traditional secondary school classrooms and finally identifies eight categories of emotions: attentive, confused, tired, bored, distracted, silent, nervous, and pleasant. The emotions of each speech sample in the dataset were jointly labeled by three people, and the final emotion labels of the samples were determined by the principle of minority rule. The number of samples in each category of emotions in the dataset is shown in Figure 1. The database contains three kinds of labels: emotion learning behavior label, discrete emotion label, and dimensional emotion label (two-dimensional (arousal-valence) emotion), subjects labeled using specific emotion words, labeled learning behavior, and discrete learning expressions. The recorded videos were labeled using discrete learning emotion labels (common emotions in online learning: confused, focused, distracted, tired, happy, and bored), and the experimenter helped the subjects to examine the videos and extract the emotion clips related to academic emotions. Ensure the completeness of image sequences, the consistency of similar emotion annotations, and re-tagging for images that do not meet the conditions. By combining previous articles and comparing with the latest research, the article finally identified 8 classroom recognition emotions of concentration, confusion, exhaustion, boredom, distraction, silence, tension, and pleasure.

Because of the flexible nature of human facial expression, the change of expression is nonrigid, and considering the requirement of real-time detection of multiple targets, this study will use the multi-task convolutional neural network (MTCNN) proposed in 2016 in the part of implementing face detection. It has very good experimental and application effects, MTCNN uses three different depths of network types to achieve face detection, and its cascade structure + CNN + bounding box method can achieve real-time face detection, which can better meet the task of multiple face detection in classroom scenes. The captured video is fed into a multi-task convolutional neural network (MTCNN), which detects the faces obtained from the video in real time and locates the key parts of the face: eyes, nose, and mouth. This model mainly uses three cascade networks, P-Net, R-Net, and O-Net, to detect and locate faces more accurately by stepwise fine-tuning.

The original face images have the problem of pose and scale differences due to different shooting conditions. Geometric normalization includes face correction and scale scaling, which can correct the angularly shifted faces and scale the faces of different scales to a uniform scale for subsequent dataset construction and use. Face correction is based on the angular difference between the original face pose and the standard face pose, and the original image is corrected to the standard face pose by coordinate rotation to ensure the consistency of face orientation. The center coordinates of the left and right eyes in the intercepted face image are obtained by feature point detection, the angle between the line connecting the center coordinates of the two eyes and the horizontal direction is calculated, and the original image is rotated by coordinates with the center of the line connecting the center coordinates of the two eyes as the rotation center to obtain the orthographic image.

(4) $$\begin{array}{c} \theta =\arccos\frac{y_{\text{right}}-y_{\text{left}}}{x_{\text{left}}+x_{\text{right}}}. \end{array}$$

The original face images often have different face sizes and scales due to differences in shooting locations and individual differences. Through scale normalization, the faces in the original images are scaled to a uniform standard to reduce or even eliminate noise interference, making the accuracy of the subsequent expression recognition algorithm evaluation more reliable. Scale normalization includes cropping and scaling of the corrected face images. The feature points of the corrected face image are calculated according to the previous (4) as the distance *d* between the two eyes, the center of the line connecting the two eyes is used as the origin, the area with distance *d* is cropped to the left and right, and the area with distance *d* and 1.5 *d* is cropped upward and downward, respectively, to obtain the standard face rectangular area. The intercepted standard face area is scale transformed to a uniform size by bilinear interpolation to achieve normalization in scale. This paper realizes the prediction of dimensional emotional data based on related networks such as recurrent neural network and finally uses the quantitative algorithm of emotional eigenvalues to calculate emotional intensity, which provides a database for the system to perform adaptive adjustment.

## 4. Classroom Emotion Recognition Model Design

To achieve a deeper network to extract deeper features while reducing the network parameters and improving the computational efficiency, a bottleneck architecture is used for the residual unit. A 1 × 1 convolutional kernel is used at the beginning and the end, and only a 3 × 3 convolutional kernel is used in the middle. The information transfer in the residual cell consists of two types of mapping, namely, residual mapping and constant mapping. The mapping generated by the convolutional and activation operations is called residual mapping; at the same time, it allows the direct transfer of information between residual units in the form of jump connections, and the mapping generated by this direct connection is called constant mapping, which is directly used as the input of the next residual unit and propagated from the top to the bottom layer. In traditional neural networks, some information is lost when the information is passed in the convolutional and fully connected layers. The information transfer between residual units can be expressed as the following equation.

(5) $$\begin{array}{c} x_{l+1}=x_{l}+f\left(x_{l}\right)\times f\left(w_{l}\right), \end{array}$$

*x*_*l*_ denotes the constant mapping, *f* denotes the residual mapping, and *w*_*l*_ denotes the convolution kernel parameter. From this equation, the output of any layer can be represented by the output of any layer shallower than it and the sum of the residuals between the two layers. The input image is passed through the deep residual network, and the final output is obtained as the feature representation *F* ∈ *R*^*W*×*H*×*C*^ of the overall image feature.

For the training set (*x*_*i*_, *y*_*i*_)=1, where *x*_*i*_ denotes the sentiment image samples, *y*_*i*_ ∈ {1,2,…, *n*} like the sentiment labels corresponding to the samples, N denotes the number of image samples in the training set, and *k* denotes the number of sentiment categories. For each sentiment image sample, the overall feature, i.e., the output of the last convolutional layer of the deep convolutional neural network, is *F* ∈ *R*^*W*×*H*×*C*^. For the feature map of each channel, the richer the sentiment information contained in a location, i.e., the more strongly the location expresses the sentiment, the larger the value of that location in the feature map. The sentiment activation map is then generated by CAM using the image-level sentiment labels.

(6) $$\begin{array}{c} V_{i}=\left(WH\right)\sum_{n=1}^{m}f\left(m,n\right),\\ \text{frature}=\sum_{i=1}\varpi_{i}\cdot \text{sum}\left(r_{i}\right)+N. \end{array}$$

**Figure 2:**
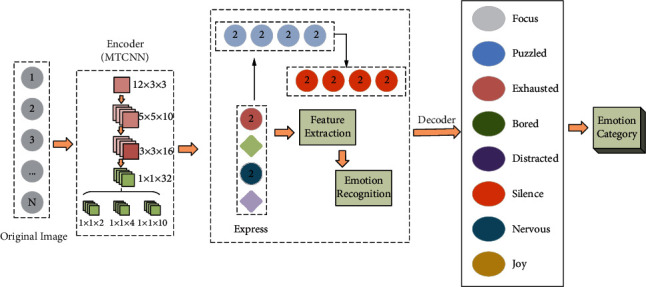
Classroom student emotion recognition algorithm.

The process of emotion recognition algorithm is mainly divided into three steps: face detection, feature extraction, and emotion recognition. The original image to be detected first needs to be preprocessed, and the preprocessing includes some digital image processing operations on the original image and face detection operations. Digital image processing operations such as histogram equalization are used on the original image to make it easier to detect and extract faces in the face detection process [19]. Since the existing images for emotion recognition are of high quality and face detection is the most important part of the preprocessing process, most of the literature related to emotion recognition uses face detection as preprocessing. Next is the feature extraction process, where the acquired faces are extracted by feature extraction methods to characterize the current facial expressions. Finally, the emotion classification is performed, and the emotion features extracted by the feature extraction algorithm are classified by existing classification methods to give the corresponding emotion list labels of the input face images. Compared with the traditional emotion recognition process, for a specific classroom environment, it is necessary to first obtain the real-time video of the classroom teaching process through the camera, use tools such as OpenCV to obtain each frame of the acquired real-time video as the original image, and then carry out the traditional emotion recognition process to get the corresponding emotion category labels of the input face images. It is necessary to analyze the emotions of the classroom students identified in a period. Finally, we need to analyze the classroom students′ emotions identified in a period and provide timely feedback to the teacher, to help the teacher adjust the teaching strategy. The classroom student emotion recognition algorithm designed in this paper is shown in Figure 2.

In recent years, an increasing number of researchers have started to combine deep learning techniques with visual sentiment analysis. The performance of a deep learning model is largely determined by the structure of the deep network, and therefore, deep learning models with different structures are often designed and employed to suit different tasks. In this section, classical deep learning models are introduced, with a particular focus on convolutional neural networks, which are widely used in visual sentiment analysis tasks. The partial connection method it uses can effectively avoid the redundant data problem caused by the full connection. And in the convolutional neural network, its local connection method can effectively reduce the amount of parameters of the network, which can reduce the dependence of the model on the huge amount of training data.

Visual feature extraction mainly uses computer vision-related technologies and digital image processing-related technologies to extract visual features closely related to human emotions and moods from image and video data according to the psychological and physiological characteristics of human beings. According to the level of the extracted features, the features can be classified into three categories: bottom-level features, middle-level features, and top-level features. Traditional machine learning methods rely more on manual design and extraction of features when processing raw data, which makes traditional machine learning algorithms have great limitations. Compared with traditional machine learning algorithms, deep learning enables automatic learning of robust features from large-scale data, abandoning complex feature engineering, and at the same time, deep learning techniques have strong domain adaptation and model generalization capabilities. Due to the massive data available on the Internet and the development of computer hardware, deep learning techniques are widely used in various fields.

**Figure 3:**
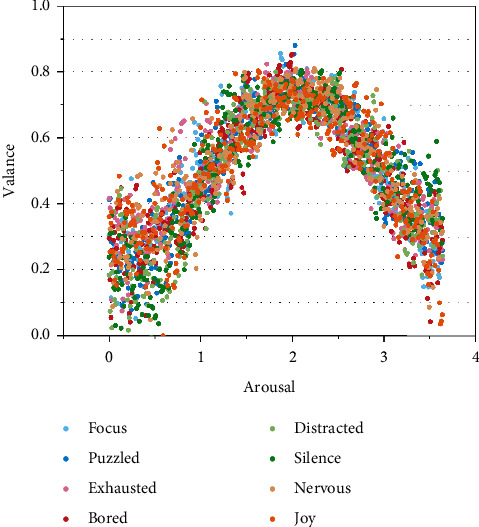
Spatial distribution of the 8 emotions in the arousal-valence dimension.

This section focuses on a qualitative approach to demonstrate the reliability of the labeled reporter labeling results, and the arousal-valence emotion model can be used to better identify students′ emotions during the learning process compared to the discrete emotion model. As shown in Figure 3, the distribution of the eight emotion categories in the arousal-valence space shows that (1) a single emotion (e.g., pleasant) can lead to multiple arousal-valence values [20]. This suggests that each category of emotion may have a different arousal-valence distribution, which means that traditional discrete emotion categories may not accurately describe a person′s internal emotions. (2) There is overlap between emotions, suggesting that different emotion categories may have similar arousal-valence distributions. For example, the arousal, valence values of some “focused” and “pleasant” images are very close to each other. This suggests that each person has a different understanding of linguistic features. In terms of description, the consistency of human categorization markers for emotions is quite poor. As can be seen, it is not easy to choose an emotion from many clear words to describe a person′s emotion, because there are nuances between some emotion labels or relationships between emotions.

To provide a benchmark for automatic emotion classification on the database (SHZ-LSD), this paper validates the recognition based on convolutional neural network and recurrent neural network database contents, while the best model for emotion recognition is used in the adaptive learning system to build the emotion recognition module of the student model. The experiments are divided into emotion recognition for discrete emotions and emotion recognition for two-dimensional arousal-valence. Discrete emotion recognition uses a convolutional neural network, the experiments are performed on the original image data for face cropping, to increase the generalization ability of the model, data augmentation is used, and the leaky ELU activation function is used for the experiments to get the best model; compared with discrete emotion, dimensional emotion data can not only reflect the spatial information of the data but also describe the temporal information of the data. To this end, this paper implements the prediction of dimensional sentiment data based on recurrent neural networks and other related networks, and finally uses a quantitative algorithm of sentiment eigenvalues to calculate the sentiment intensity, which provides the database for the system to perform the adaptive adjustment.

## 5. Classroom Emotion Recognition Model Performance Test Results

In this chapter, the global features extracted by IS10 and IS13 are screened using FC for better feature fusion, and the experimental results show that the combined features (IS10 + IS13) of global features extracted by IS10 and IS13 work best compared with the global features extracted by each of IS10 and IS13 [21]. The temporal features were also extracted with IS10 and IS13 and screened using 1D-CNN, and the experimental results showed that the combined features (IS10IId + IS13lld) with temporal features extracted by IS10 and IS13 worked better compared with MFCC. Therefore, the last dense layer output of (IS10 + IS13) in FC is used as the global optimal feature, and the last dense layer output of (IS10Ild + IS131ld) in 1D-CNN is used as the temporal optimal feature. To reduce the correlation between global and temporal features, the CoreNet network is used to fuse the filtered global optimal features and temporal optimal features to obtain fused features, which are trained on FC, and the results show that the results of fused features based on CoreNet are optimal, thus proving the feasibility of the method. One of the applications of artificial intelligence in education is to monitor valuable information such as students′ learning expressions and learning postures in real time, pay attention to each student′s emotional state, and make corresponding adjustments for feedback, and teachers adjust the rhythm of the classroom according to the specific situation. Classroom efficiency will be significantly improved.

**Figure 4:**
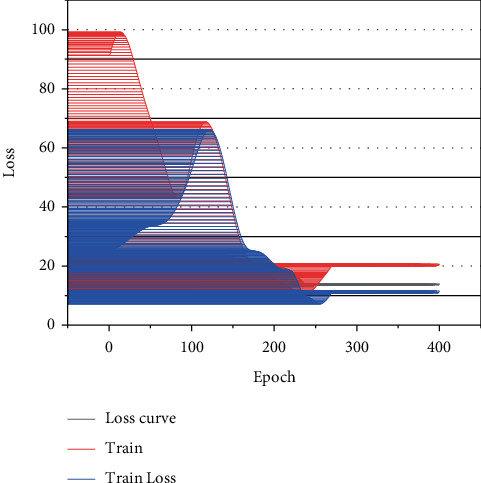
Change of loss value during training.

In this study, a single ID photo of 100 people is selected as the original sample and a small sample dataset is constructed. Because the ID photos have the same background, it can make the faces in the images at the same angle, which can eliminate the influence of different image backgrounds and avoid the influence of face skin color, etc. Based on this, data enhancement techniques such as mirror transformation, multi-region cropping, Gaussian noise, symmetric expansion, and bit-plane method are carried out to expand the sample database [22]. To achieve expression recognition, we use adversarial generative networks to expand the samples, extract expressions from expression feature samples, and use convolutional neural network models to “attach” them to a single face image and train them to generate face images with different expressions. The loss test is shown in Figure 4, which shows that the loss value decreases and smooths out as the number of iterations increases.

**Figure 5:**
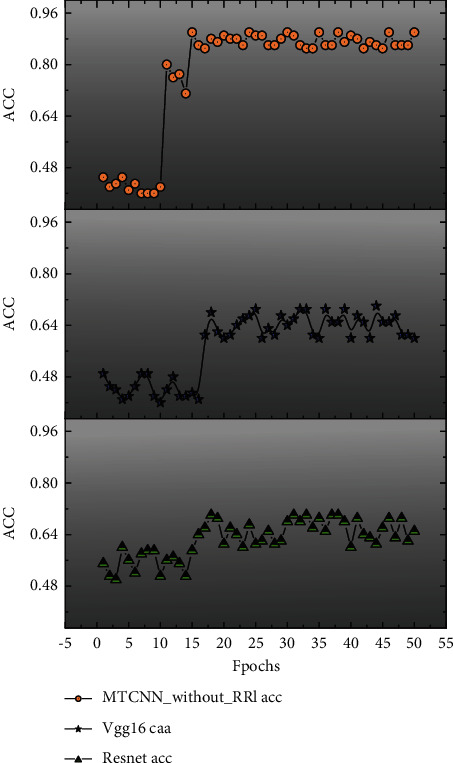
MTCNN accuracy comparison chart.

To further verify the effect of the representative region loss function designed in this paper on the model recognition, the MTCNN without RRL was tested in RAF-DB with added feature images. The accuracy of the MTCNN without RRL on the RAF-DB test set is 76.5%, which compares with that of the pretrained model in Figure 5. The blue curve represents the test accuracy of the MTCNN model without RRL, which nearly overlaps with the accuracy curves of ResNet18 and VGG16 in the fitting part, indicating that the MTCNN model without RRL has the same recognition effect as the two pretrained models without RRL. The difference between the recognition results of the MTCNN model without RRL and the two pretrained models is small.

The MTCNN model with RRL has a faster fitting speed with the same training parameters and achieves the fit in the 15th round, while the model without RRL has oscillations in the test set, a slow fitting speed, and relatively low accuracy [23]. Therefore, RRL can effectively improve the performance of the MTCNN model and has an improvement effect on the recognition of the MTCNN model.

**Figure 6:**
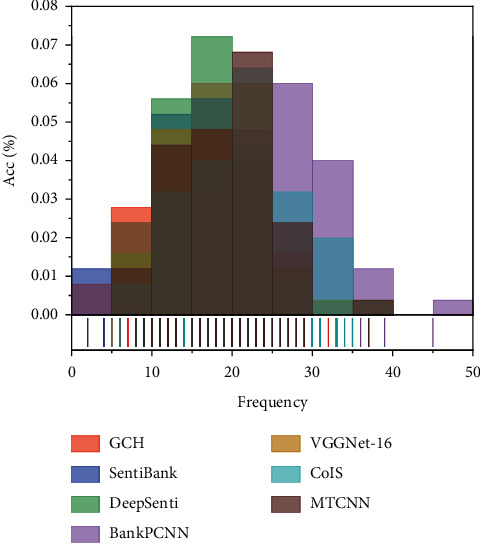
Classification accuracy of different methods on the Emotion ROI dataset.

The proposed method in this paper was evaluated on three datasets Twitter I, Twitter II, and Emotion ROI to demonstrate its effectiveness. The three datasets were divided into 80% training set and 20% test set using random partitioning. The proposed method achieves 79.83% and 78.25% classification accuracy on Twitter I and Twitter II datasets, respectively, which are higher than the traditional visual sentiment analysis method GCH and Sent bank based on intermediate semantic representation [7]. The deep learning-based visual sentiment analysis methods outperform the traditional visual sentiment analysis methods in terms of performance. The classification results of the proposed methods in this paper are improved compared with the comparison methods on both datasets, and the COIS model with the best comparison results has improved the classification accuracy by 0.93% and 1.42% on the two datasets, respectively. The classification results of the test method and the five comparison methods on the meta classifier sentiment image dataset Emotion ROI are evaluated in terms of accuracy %, and the accuracy comparison is shown in Figure 6.

The proposed method achieves 49.34% classification accuracy on the multi-category sentiment image dataset Emotion ROI, which is more accurate than the traditional visual sentiment analysis method GCH and the intermediate semantic representation-based visual sentiment analysis method Sent bank. The classification accuracy of DA-MLCNN is 6.81% and 3.88% higher than that of DeepSentiBank and VGGNet-16, and 1.78% and 1.21% higher than that of PCNN and COIS models, respectively. By comparing the classification results of the various methods on the multiclassification dataset, it can be shown that the DA-MLCNN method proposed in this paper can also be adapted to the multiclassification task of visual emotion [6]. The combined classification performance on both dichotomous and meta classifier sentiment image datasets shows that the proposed method can learn more discriminative visual features and thus improve the visual sentiment analysis.

**Figure 7:**
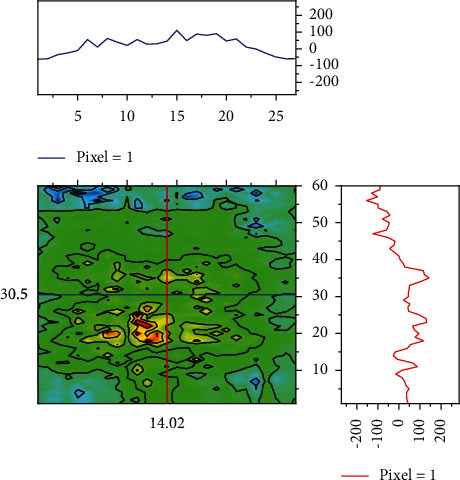
Joint probability distribution of parameters.

Through scale normalization, the face in the original image is scaled to a unified standard to reduce or even eliminate noise interference, which makes the evaluation accuracy of the subsequent expression recognition algorithm more reliable. First, a class is sampled and analyzed as Classroom A. Classroom A is analyzed by running the program. The *X*-axis represents the values of the parameters, and the corresponding *Y*-axis represents the corresponding posterior probabilities; if the posterior probabilities are larger, then the probability that the parameters *μ* and*σ* are the true values will be larger [3]. In such a plot, it is intuitive to obtain more reasonable values from the posterior. It can also be seen that the curves of the parameters in the left plot are smoother, and the right plot looks like white noise, which means that there is a good degree of mixing. And the maximum posterior estimate of each variable, which is the peak in the left-hand distribution, is very close to the true parameters. As shown in Figure 7, the parameters *μ* and*σ* do not have any correlation, meaning that the two parameters are independent of each other and will not be linearly correlated. Finally, a maximum posterior density (HPD) interval is run. An HPD interval is the smallest interval that contains a certain proportion of probability density and is often used to describe the dispersion of the posterior distribution of the parameters.

When making statistical inferences, the size of the sample that can be relied upon is often limited by various conditions. Direct use of means is intuitive and simple, but the meaningfulness of statistical inference cannot be determined by the size and complexity of the computation alone. The mean value calculated directly is easily affected by the sample characteristics, the way the sample is drawn, and other factors, especially when the sample size is limited, making it more difficult to make reasonable inferential statistics. Using the Bayesian probability model for statistical analysis, the MCMC under the framework of the PyMC3 probability model estimates the posterior distribution of parameters by random sampling, and then the highest probability value in the posterior distribution is used as the estimator of the parameters, the more the samples, the more the posterior distribution converges, and the resulting estimates will be closer to the real situation.

## 6. Conclusion

In this paper, we perform face detection and image segmentation with the help of a multi-task convolutional neural network (MTCNN), select the ones with better feature morphology to build a standard database, and further improve and build a classroom-based facial expression classification standard. A visual motion analysis method with the fusion of overall and local features of images is proposed. The method designs a multi-scale full convolutional neural network for detecting the salient regions of images and extracting the features of the salient regions of images, while generating class activation mappings of emotional images using only the image-level emotional labels, and finally generating emotional activation maps of images and extracting the features of emotional regions by superimposing class activation mappings of multiple emotional categories. Suitable parameters are designed according to the characteristics of the experimental data, and the constructed dataset is trained. After testing and analysis, the results show that the model of constructing a task convolutional neural network performs well on the student classroom facial expression dataset, and the recognition rate using the test set is at 91%, which indicates that the method is scientifically feasible and can reduce the reliance on the training sample size, reduce the collection workload, and alleviate the pressure of data storage. However, due to the small sample size of the classroom emotion dataset constructed in this paper, the emotion labels are representative but incomplete and cannot fully characterize the recognition effect of the model designed in this paper. For the construction of the emotion dataset in a classroom environment, future work, other emotion categories, or even compound emotion categories can be added to further expand the sample size of the dataset. Increase the number of labelers to further enhance the accuracy of the dataset picture labels.

## Data Availability

The data used to support the findings of this study are included within the article.
